# Dendrite-scale quantitative evaluation of solidification cracking in welding using in-situ synchrotron X-ray imaging: distribution characteristics of critical strain

**DOI:** 10.1080/14686996.2026.2736326

**Published:** 2026-10-12

**Authors:** Houichi Kitano, Tomoya Nagira, Fumiyoshi Yoshinaka, Takahiro Sawaguchi, Takayuki Yamashita, Yasuhiro Aoki, Hidetoshi Fujii

**Affiliations:** a Research Center for Structural Materials, National Institute for Materials Science, Tsukuba, Ibaraki, Japan; b Joining and Welding Research Institute, The University of Osaka, Ibaraki, Osaka, Japan

**Keywords:** Solidification cracking, in-situ synchrotron X-ray imaging, dendrite-scale observation, critical equivalent strain, critical-strain distribution, welding

## Abstract

Solidification cracking is a critical defect in welding, and its occurrence has conventionally been described using temperature-dependent critical-strain curves. However, whether the critical strain is uniquely determined by temperature remains unclear, particularly at the dendrite scale. In this study, solidification-cracking behavior in an Fe–15Mn–10Cr–8Ni–4Si alloy was investigated using in-situ synchrotron X-ray transmission imaging combined with local strain and temperature analyses. Crack-initiation events were identified from the time-resolved X-ray images, and the corresponding critical equivalent strains and critical temperatures were extracted at individual positions along the crack path. Solidification cracking occurred over approximately 1320–1000°C, corresponding to the effective solid–liquid coexistence region predicted by a Scheil–Gulliver calculation. The median critical strain was higher at lower temperatures and generally decreased toward higher temperatures. A broad critical-strain distribution remained within each 30°C temperature range. After subtraction of the temperature-bin median, the remaining variation was spatially nonuniform along the crack path. Comparison with a non-cracking case showed that most equivalent-strain values remained below the critical-strain distribution obtained in the cracking case, although the upper tail partially overlapped its lower part. These results provide experimental evidence that the critical strain at solidification-crack initiation is better represented as a temperature-dependent distribution than as a single-valued curve. The physical factors governing the distribution width and spatial variation remain to be identified.

## IMPACT STATEMENT

Dendrite-scale observations show that critical strain for solidification-crack initiation is better represented as a temperature-dependent distribution with spatially nonuniform variation than as a single-valued curve.

## GRAPHICAL ABSTRACT

**Figure:**
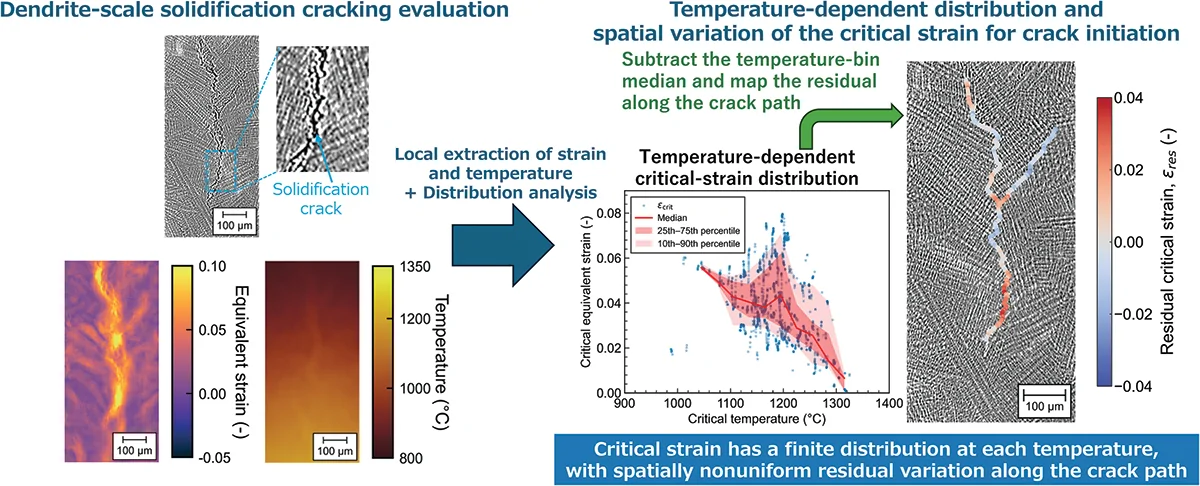

## 1. Introduction

As one of the most fundamental joining technologies in manufacturing, welding is widely used in various fields such as transportation systems and infrastructure. A major advantage of welding is its ability to efficiently integrate structural components through localized melting. However, welding is a highly non-equilibrium process involving rapid heating and cooling and it is prone to creating defects such as residual stress, distortion, cracking, and porosity [1–4]. Among these defects, solidification cracking is one of the most critical issues that significantly reduces the reliability of welded structures. It generally occurs when low-melting liquid films that remain in the final stage of solidification cannot withstand the contraction of the surrounding solidifying material owing to solute segregation [1,5]. Conventionally, the conditions for solidification cracking have been described using a critical strain curve (or a high-temperature ductility curve) in the temperature–strain space. In this framework, cracking occurs when the applied strain during cooling exceeds a critical value. Because this critical strain decreases significantly within the solid–liquid coexistence temperature range (that is, between the liquidus and solidus temperatures), solidification cracking is believed to occur preferentially in this temperature range [6]. However, such interpretations based on critical strain curves are primarily derived from macroscopic test results, and their relationship with local microstructural states and deformation behavior at the dendrite scale remains unclear. This is primarily because solidification cracking occurs locally at high temperatures and propagates rapidly, making it extremely difficult to directly observe the microstructural, strain, and temperature states at the moment of crack initiation with sufficient spatial and temporal resolution. Conventional experimental methods, such as the Varestraint test, Trans-Varestraint test [7–9], and U-type hot cracking tests [10], have enabled the evaluation of macroscopic deformation behavior and cracking susceptibility; however, they do not provide sufficient insight into crack initiation mechanisms at the dendrite scale. Owing to this lack of direct, quantitative observations at the relevant spatial scale, researchers have not adequately addressed whether the critical strain is uniquely determined by temperature or varies depending on local microstructural conditions.

Based on the above background, the present study re-examined the conditions for solidification cracking, which have conventionally been interpreted based on macroscopic test results, by using direct observations at the dendrite scale to clarify their intrinsic characteristics. Specifically, our previously developed in-situ observation techniques [11–15], combining high-brightness synchrotron X-ray transmission imaging for microstructural and strain field visualization and visible-light imaging for temperature evaluation [15], were employed. Building on Ref [15]. which established the image-based strain-evaluation method, and Ref [16]. which clarified the solidification behavior and segregation-induced extension of the solid–liquid coexistence range in this alloy, the present study focuses on the local conditions at crack initiation and the distributional characteristics of the critical strain along the crack path. The timing of crack initiation was identified from the temporal evolution of the X-ray transmission images, and the corresponding local temperature and strain at each position were quantitatively extracted. The extracted critical strains were directly characterized as a function of temperature using temperature-bin medians and percentile ranges. In addition, the temperature-dependent median was subtracted from the critical strain at each position, and the resulting residual was evaluated along the crack path to examine spatial variation not represented by the central temperature-dependent tendency. In addition, by comparing the temperature–strain histories in a non-cracking case obtained under identical welding conditions, the relationship between the extracted distribution characteristics of the critical strain and the non-cracking behavior was examined.

These analyses were conducted to determine whether the critical strain at solidification-crack initiation can be represented by a single temperature-dependent relationship or should instead be treated as a distribution with a finite width. The spatial variation of this distribution was also examined without attributing it to a specific thermal, mechanical, or microstructural factor.

## 2. Methods

In this study, an Fe–15Mn–10Cr–8Ni–4Si alloy (hereafter referred to as FMS alloy) was employed. This alloy, originally developed for damping applications, is known to be susceptible to solidification cracking during welding, making it a suitable model material for investigating the fundamental characteristics of solidification cracking behavior. An experimental and image-analysis-based approach was employed to simultaneously observe the microstructural evolution and evaluate the strain and temperature fields during welding at the dendrite scale. This section describes the experimental procedure and the evaluation methods for each physical quantity. The fundamental observation system and analysis algorithms are based on our previous work [15], and they are integrated here for the quantitative evaluation of solidification cracking conditions.

### 2.1. Experimental procedure

A schematic illustration of the in-situ observation system is shown in Figure 1 [15]. Experiments were conducted at the BL20XU beamline of SPring-8 using synchrotron X-rays with an energy of 28 keV. The specimen dimensions were 50 mm × 50 mm × 0.2 mm, forming a thin plate suitable for X-ray transmission imaging. During gas tungsten arc (GTA) welding, the transmitted X-rays were recorded using a two-dimensional detector (imaging detector, Hamamatsu Photonics, Japan) to obtain in-situ images of the microstructural evolution. Simultaneously, visible-light images were captured using a high-speed camera (NAC Image Technology, Japan) and converted into temperature maps using the two-color method. The welding conditions were as follows: a welding current of 6 A, a welding speed of 10 mm/s, and pure Ar shielding gas at a flow rate of 8 L/min. Under these conditions, two datasets were obtained: one with solidification cracking (Case 1) and one without cracking (Case 2). Although the nominal welding conditions were identical, slight differences in the mechanical constraint imposed by the fixture may have contributed to the presence or absence of cracking. Because the restraint condition was not measured independently in the present experiments, the strain and temperature states in the two cases were compared to examine their relationship with the observed cracking and non-cracking behavior. X-ray transmission images were acquired with a frame interval of 15 ms and a spatial resolution of 0.45 μm/pixel for both cases. Visible-light images were recorded at 120 fps for Case 1 and 250 fps for Case 2, both with a spatial resolution of 2.1 μm/pixel. The difference in frame rate was due to equipment constraints. The X-ray transmission images were acquired at approximately 66.7 fps, whereas the visible-light images were acquired at higher frame rates in both cases. As described later, the visible-light temperature data were temporally interpolated to the acquisition times of the X-ray images and spatially resampled to the X-ray image grid. Therefore, the temperature fields in both cases were evaluated on the same temporal and spatial grid, although temperature variations occurring faster than the original visible-light acquisition rate could not be recovered.

**Figure 1.**
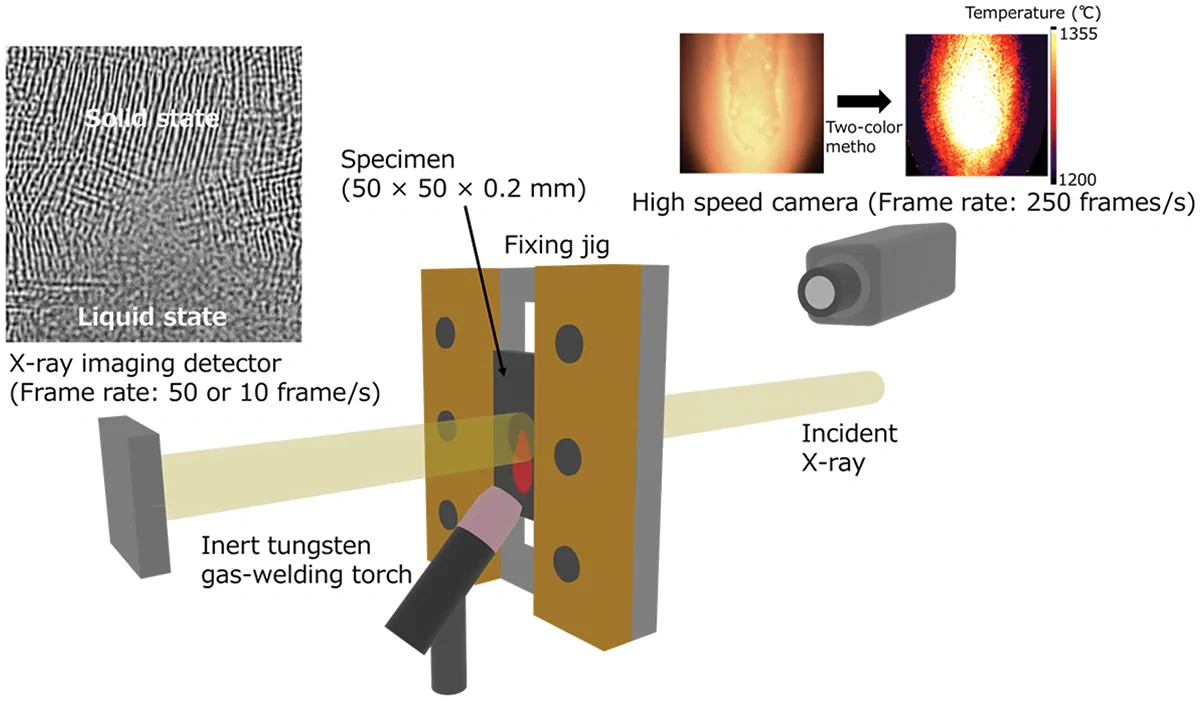
Schematic of the in-situ observation system [15]. Thin-plate specimens were joined by gas tungsten arc (GTA) welding under synchrotron X-rays at SPring-8 BL20XU. Transmitted X-rays were recorded with a 2D detector, while visible-light images were captured with a high-speed camera and converted to temperature maps by the two-color method.

### 2.2. Evaluation method

#### 2.2.1. Strain evaluation method

The strain distribution was evaluated following the procedure shown in Figure 2(a) based on our previous work [15]. The processing pipeline consists of patch splitting, denoising using a trained neural network, patch merging, patch splitting, contrast correction using another trained neural network, patch merging, patch splitting, TV-L1 optical flow computation, patch merging, and strain calculations. The patch splitting and merging procedures are illustrated in Figure 2(b).

**Figure 2.**
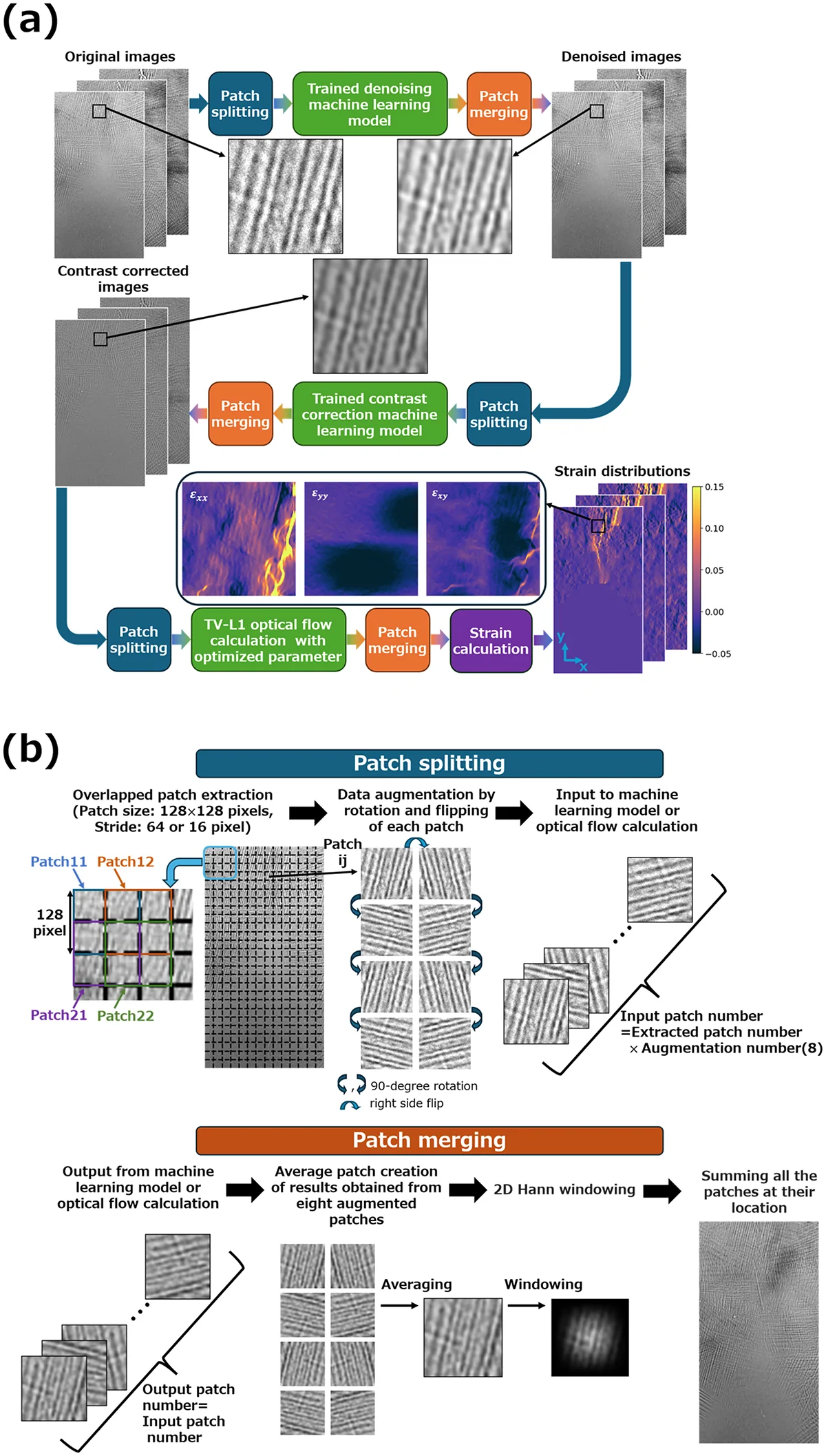
Procedure for strain field evaluation from X-ray transmission images using denoising, contrast correction, and TV-L1 optical flow analysis [15]. (a) Overview of the strain evaluation method. (b) Patch splitting and merging procedures. The patch stride was 64 pixels for the denoising and contrast correction procedures and 16 pixels for the TV-L1 optical flow analysis.

Convolutional neural network (CNN)-based architectures were employed for denoising and contrast correction (Figure 3). For the denoising network, the loss function was defined as the L2 loss between the output image and a reference image generated by applying a donut-shaped smoothing kernel to randomly selected pixels. For the contrast correction network, the loss function was defined as a combination of the image entropy (entr) and the structural similarity index (SSIM) [17], as expressed in Eq. (1).

(1) $$\mathrm{los}s_{\mathrm{cont}}=\frac{1}{n}\sum \left(\mathrm{entr}+\left(1-\mathrm{SSIM}\right)\right),$$

**Figure 3.**
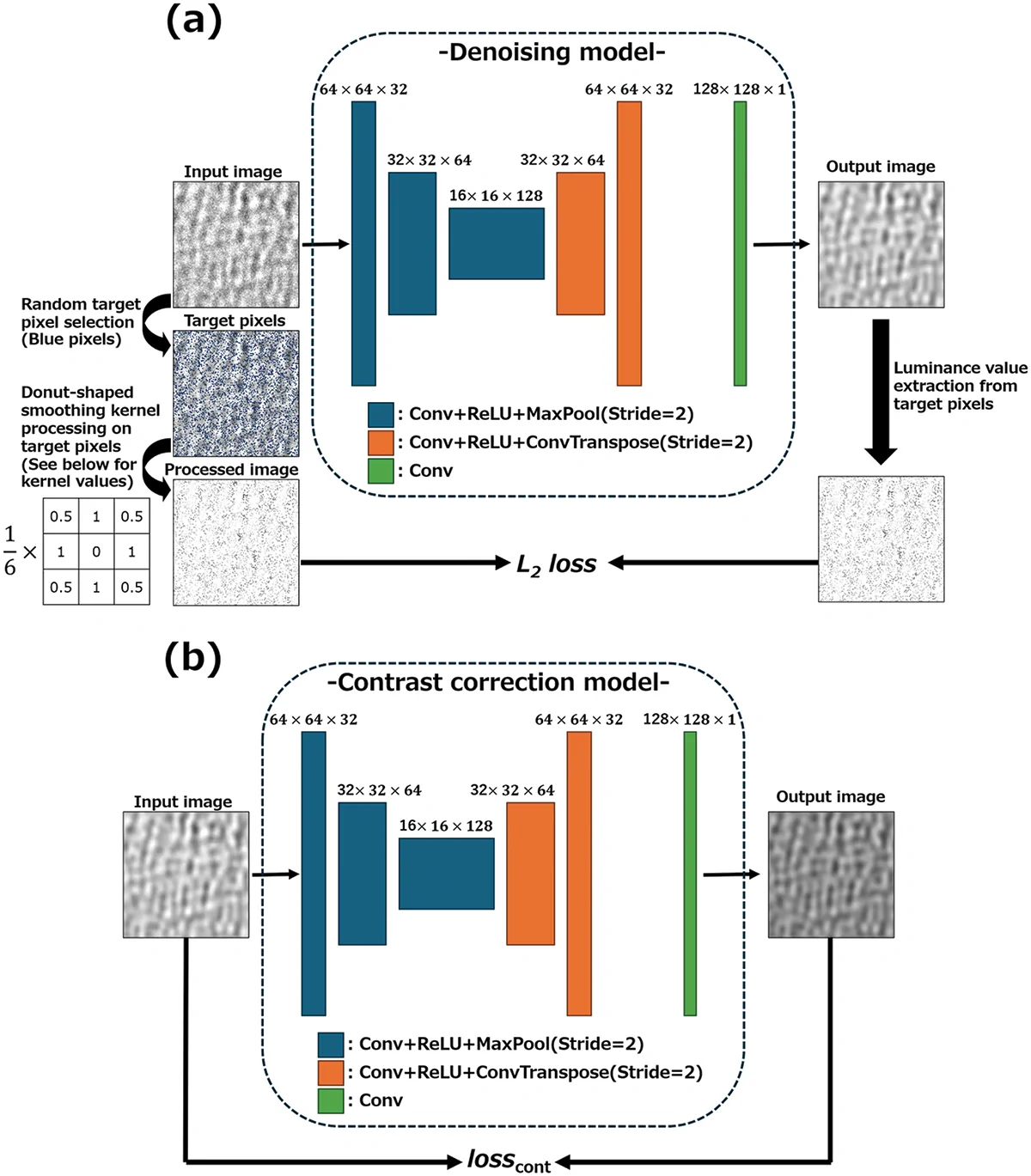
Convolutional neural network architectures for (a) the denoising model and (b) the contrast correction model [15].

where n is the number of samples. The networks were trained using the AdamW optimizer with a weight decay parameter of $10^{-5}$ and a learning rate of $10^{-3}$. Training image patches were generated by randomly extracting 128 × 128 pixel regions from the time-series images. Only patches located entirely within the solid phase were used for training. The denoising and contrast correction networks were trained separately for each case. In other words, two sets of trained models were constructed in this study, one for Case 1 and one for Case 2. To reduce the influence of extreme intensity values in the original images, the pixel intensities were clipped for each time-series dataset in each case. Specifically, intensity values outside the range of ±2σ (where σ denotes the standard deviation of the intensity distribution) were clipped to the corresponding boundary values.

Parameters of the TV-L1 optical flow, particularly the attachment parameter [18], were determined for each dataset by minimizing the discrepancy between the forward and backward displacement fields, as defined in Eq. (2).

(2) $$\mathrm{los}s_{\mathrm{disp}}=\frac{1}{n}\sum \left[\left\{\frac{{\left(u_{f}+u_{b}\right)}^{2}}{u_{f}^{2}+u_{b}^{2}}\right\}+\left\{\frac{{\left(v_{f}+v_{b}\right)}^{2}}{v_{f}^{2}+v_{b}^{2}}\right\}\right].$$

Here, $u_{f}$ and $v_{f}$ denote the pixel-wise displacement components in the x- and y-directions from time t to t+1, while $u_{b}$ and $v_{b}$ denote those from time t+1 to t, respectively. To improve the stability of strain field estimation, the patch stride was reduced to 16 pixels only in the patch-splitting step immediately before the TV-L1 optical flow computation, thereby increasing the degree of overlap between patches.

In the strain evaluation, the cumulative displacement from the initial frame to each time frame was first computed using the frame-wise displacement fields, and this was then used to derive the strain field at each time step. To compute the cumulative displacement, nodes were assigned to all pixel positions in the initial image (at time t=0), where zero strain was assumed. Let H and W denote the numbers of pixels in the vertical and horizontal directions of the image, respectively. The node located in the i-th row and j-th column is denoted as node ij. The initial coordinates of this node, $x_{ij}^{0}$ and $y_{ij}^{0}$, are defined as

(3) $$x_{ij}^{0}=j,y_{ij}^{0}=i\left(i=1,\cdots H,j=1,\cdots W\right).$$

Next, using the displacement fields in the x- and y-directions at each time frame ($u^{t}$ and $v^{t}$), the coordinates of each node at time t ($x_{ij}^{t}$ and $y_{ij}^{t}$) are computed as

(4) $$x_{ij}^{t}=x_{ij}^{0}+\overset{t}{\underset{t=1}{\sum }}u^{t}\left(x_{ij}^{t},y_{ij}^{t}\right),y_{ij}^{t}=y_{ij}^{0}+\overset{t}{\underset{t=1}{\sum }}v^{t}\left(x_{ij}^{t},y_{ij}^{t}\right).$$

Here, the displacement values $u^{t}$ and $v^{t}$ at non-integer coordinates $\left(x_{ij}^{t},y_{ij}^{t}\right)$ were obtained by cubic spline interpolation. Using these coordinates, the cumulative displacements in the x- and y-directions from time 0 to time t, denoted as $U_{ij}^{t}$ and $V_{ij}^{t}$, were calculated as

(5) $$U_{ij}^{t}=x_{ij}^{t}-x_{ij}^{0},V_{ij}^{t}=y_{ij}^{t}-y_{ij}^{0}.$$

The in-plane strain components at time t, were then computed from the spatial gradients of the cumulative displacement field as

(6) $$\varepsilon_{xx}^{t}=\frac{\partial U^{t}}{\mathrm{\partial x}},\varepsilon_{yy}^{t}=\frac{\partial V^{t}}{\mathrm{\partial y}},\varepsilon_{xy}^{t}=\frac{1}{2}\left\{\frac{\partial U^{t}}{\mathrm{\partial y}}+\frac{\partial V^{t}}{\mathrm{\partial x}}\right\}.$$

The spatial derivatives were computed using a five-point finite difference scheme. At solid–liquid interfaces, discontinuities in displacement were observed even after solidification; therefore, finite differences were not computed across such interfaces, and one-sided differences were used instead. One-sided differences were also applied at the image boundaries, while central differences were used in all other regions. A median filter with a window size of 11 × 11 pixels was then applied separately to each in-plane strain-component field. The von Mises-type equivalent strain, $\varepsilon_{eq}^{t}$, used in the subsequent analysis was calculated from the filtered in-plane strain components as

(7) $$\varepsilon_{eq}^{t}=\sqrt{{\left(\varepsilon_{xx}^{t}-\varepsilon_{yy}^{t}\right)}^{2}+{\left(\varepsilon_{xx}^{t}\right)}^{2}+{\left(\varepsilon_{yy}^{t}\right)}^{2}+3{\left(\varepsilon_{xy}^{t}\right)}^{2}}$$

Because the out-of-plane strain components could not be measured from the X-ray transmission images, they were set to zero solely for the purpose of defining this scalar equivalent-strain measure. The resulting equivalent strain is therefore an image-based measure derived from the in-plane components of the projected displacement field and should not be interpreted as a complete three-dimensional constitutive strain of the semisolid material.

#### 2.2.2. Temperature evaluation method

Visible-light images obtained by the high-speed camera were first denoised using the procedure described in Section 2.2.1 (patch splitting, denoising network, and patch merging), followed by temperature evaluation using the two-color method. The temperature was calculated from the red (~674 nm) and green (~589 nm) channels of the visible-light images using the two-color method. The calculations were performed using a custom implementation developed in this study. The constants used in the temperature calculations were identical to those employed in commercial two-color thermography software (Thermias, NAC Image Technology Inc.), which were determined through calibration using a blackbody furnace. The validity of the implemented method was confirmed by comparing the resulting temperature fields with those obtained from Thermias using the same visible-light images, and good agreement was observed. After calculating the temperature distribution, temporal interpolation using cubic splines and spatial interpolation using bilinear interpolation were applied to match the temporal and spatial resolutions of the X-ray transmission images. To reduce noise, median filtering was applied with a window size of 5 frames in the temporal direction and 15 × 15 pixels in the spatial direction. Finally, the surface temperature field was spatially aligned with the X-ray transmission images by referencing the observed liquid-phase geometry.

#### 2.2.3. Coordinate system unification

The strain field evaluated using the method described in Section 2.2.1 is expressed in a Lagrangian coordinate system, as it is based on tracking the displacement of each pixel from its initial position. In contrast, the X-ray transmission images and temperature fields are defined in an Eulerian coordinate system because they represent the values at fixed spatial locations. To directly compare these quantities, all data were transformed into a common coordinate system. In this study, the X-ray transmission images and temperature fields were mapped onto the Lagrangian coordinate system, using the displacement fields obtained from the optical flow analysis. Specifically, the positions of nodes defined at the initial time were updated over time using the cumulative displacement, and the corresponding temperature and transmission intensity values were obtained by interpolation at these positions. Through this procedure, the strain, temperature, and X-ray transmission data were consistently represented in the same Lagrangian framework, enabling quantitative comparison of their spatial and temporal evolutions in the vicinity of solidification cracking. It should be noted that the temperature field obtained by two-color thermography represents the specimen surface, whereas the X-ray transmission images and the displacement and strain fields derived from them represent information projected through the specimen thickness. The two fields were spatially aligned using the observed liquid-phase geometry and were compared within the common Lagrangian coordinate system described above. The small specimen thickness of 0.2 mm may reduce a thermal gradient along the specimen thickness. Nevertheless, possible through-thickness variation and exact equivalence between the measured surface temperature and the projected internal state could not be independently verified in the present experiment.

## 3. Results and discussion

### 3.1. Overview of observation results

Figures 4 and 5 present observations for the two cases with (Case 1) and without (Case 2) solidification cracking, respectively. The upper panels (a) show the contrast-corrected X-ray transmission images (displayed as inverted grayscale images), equivalent strain maps, and temperature maps. The lower panels (b) show the strain distributions along the two lines indicated in the equivalent strain maps. The X-ray transmission images and temperature fields were mapped onto the Lagrangian coordinate system using the procedure described in Section 2.2.3. The X-ray transmission images are displayed as inverted grayscale images to improve the visibility of the observed features. Qualitatively, regions with higher density and higher X-ray absorption appear brighter, whereas regions with lower absorption, such as cracks with missing material, appear darker.

**Figure 4.**
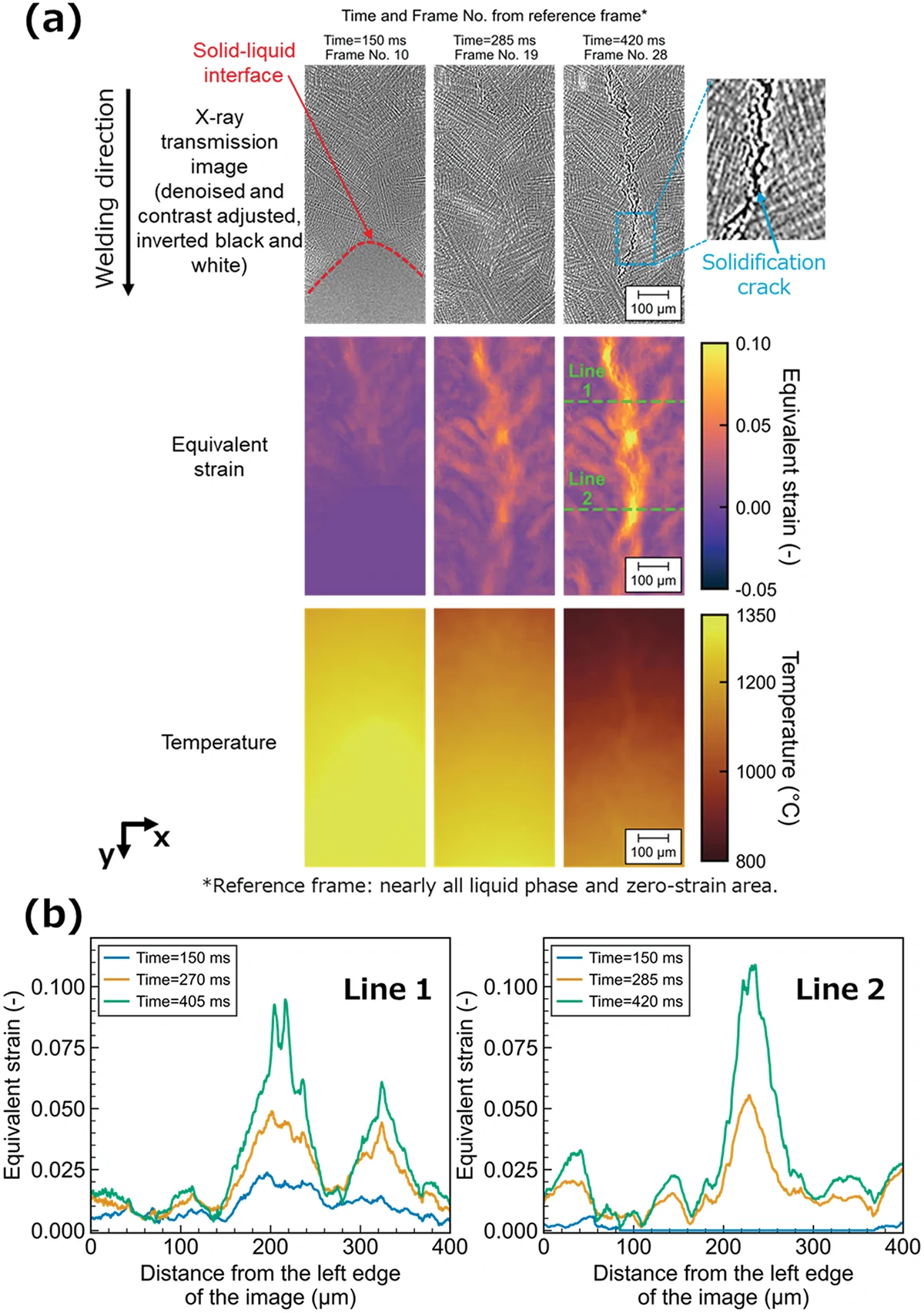
Observation results of Case 1 (with cracking). (a) Contrast-corrected X-ray transmission images (inverted black and white), equivalent strain maps, and temperature maps. (b) Line-profile strain distributions.

**Figure 5.**
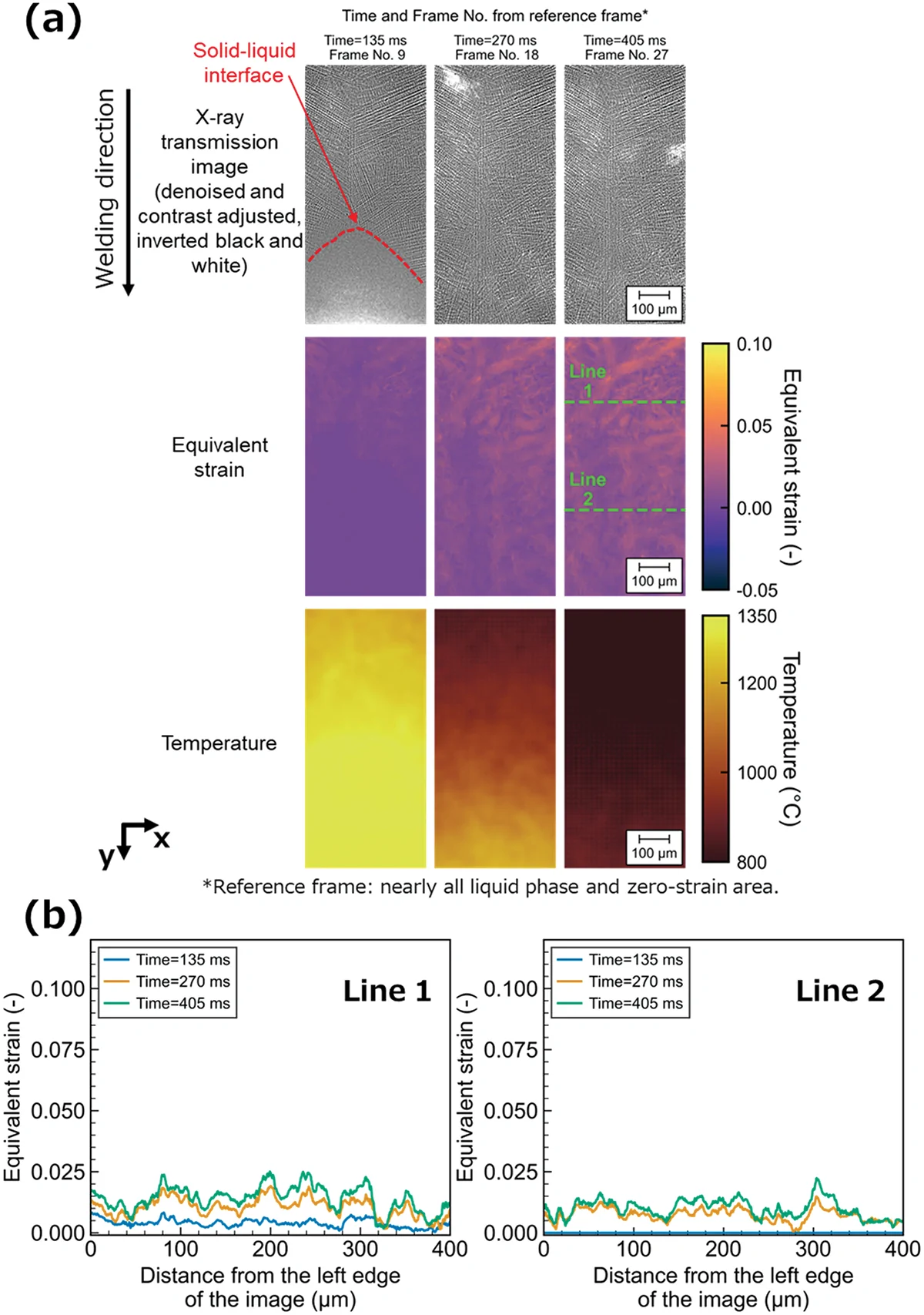
Observation results of Case 2 (without cracking). (a) Contrast-corrected X-ray transmission images (inverted black and white), equivalent strain maps, and temperature maps. (b) Line-profile strain distributions.

In Case 1, a continuous dark region corresponding to crack formation was clearly observed in the X-ray transmission images, primarily near the weld centerline, that is, in the final solidification region (Figure 4). As will be discussed in detail later, the crack formed at high temperatures of approximately 1000–1350°C, indicating solidification cracking. The corresponding equivalent strain distribution reveals pronounced local strain concentration in the crack initiation and propagation regions. In contrast, in Case 2, no continuous dark regions corresponding to cracks were observed in the X-ray transmission images, and no significant strain concentration was detected (Figure 5). Although regions with slightly higher temperatures were locally observed near the crack initiation sites in Case 1, in both cases the temperature distribution exhibited an approximately monotonic decrease over time, and there was no clear difference between the cases with and without cracking.

However, a comparison of the strain profiles along the selected lines reveals a significant difference between the two cases. In Case 1, a strong localized strain concentration appears in association with crack formation, reaching a maximum value of approximately 12%. By contrast, the strain in Case 2 remains below approximately 2.5% along both lines. Although the material and nominal welding conditions were identical, the origin of the different strain behavior between the two cases could not be determined from the present measurements. Differences in the mechanical constraint imposed by the fixture may be a cause, but the restraint condition was not independently quantified.

### 3.2. Crack formation behavior inferred from local X-ray transmission images

Figure 6 shows the overall X-ray transmission image of Case 1 (corresponding to the rightmost image in Figure 4(a)) and the temporal evolution of the magnified X-ray transmission images in a selected rectangular region. A continuous dark region was formed in the left part of Frame Nos. 18 and 22, indicating the initiation of solidification cracking. Furthermore, from Frame No. 24 onward, bright regions appear around the cracks. We consider these bright regions to correspond to locally thickened material compared with the surrounding area, which is likely caused by redistribution of the remaining liquid phase originally at the crack initiation site as the crack opens. These observations are consistent with the conventional understanding of solidification cracking, in which cracks form due to the opening and rupture of residual liquid films.

**Figure 6.**
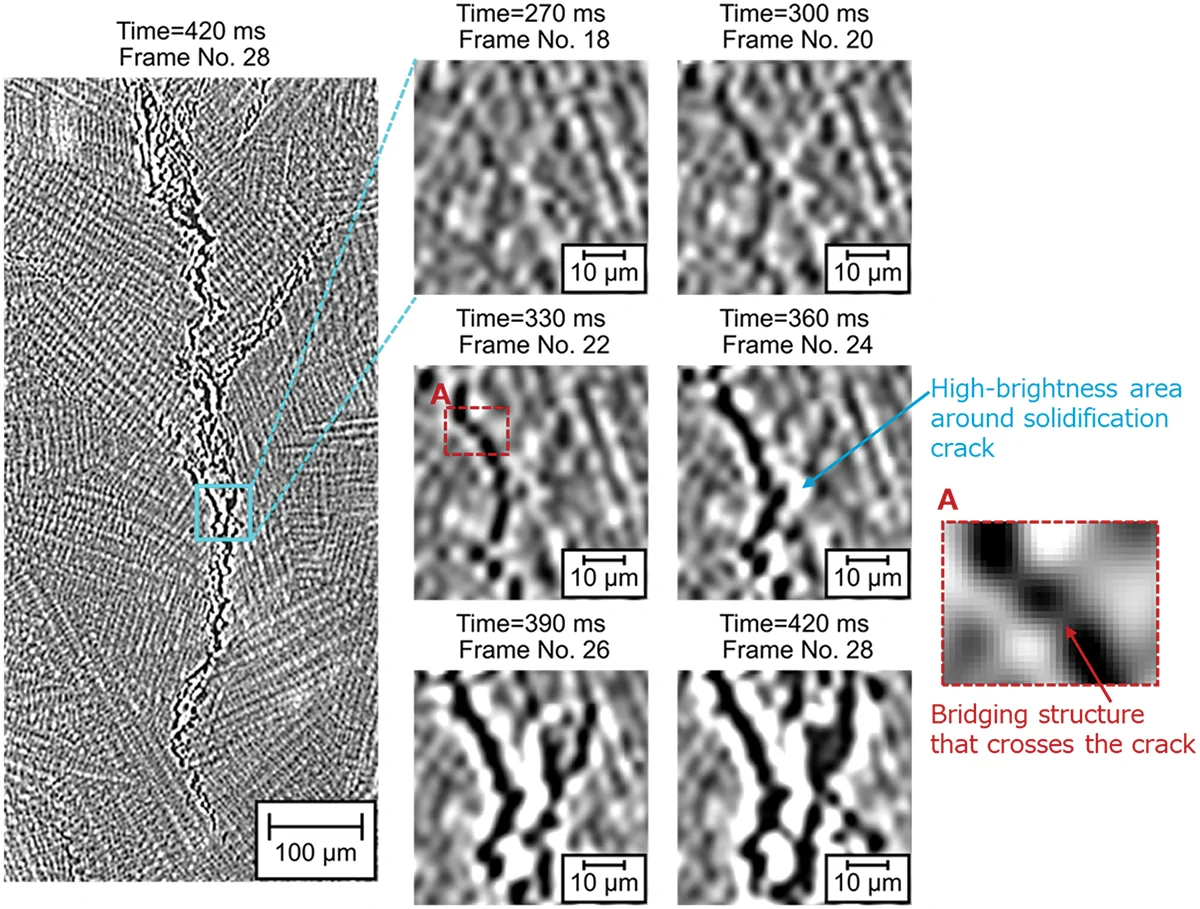
In-situ X-ray transmission images showing the dendrite-scale evolution of solidification cracking in Case 1. Left image: the overall view corresponding to Figure 4(a). Right panels: magnified views of the selected region over time (frame Nos. 18–28). Crack initiation is identified by the formation of a connected low-brightness region. Subsequently, a high-brightness area appears surrounding the crack. A bridging structure crossing the crack occurs in frame No. 22, as highlighted in region A.

In contrast, two characteristic behaviors that have not been explicitly considered in conventional theories of solidification cracking were observed. The first feature, clearly shown in Frame No. 22 and highlighted in the magnified view of region A, is the presence of a bridging structure across the crack. While conventional theories often assume the rupture of a continuous liquid film, the present observation suggests that even within regions containing low-melting residual liquid, dendrites can grow and connect to form local solid bridges. Such bridging structures may help transmit stress and strain across the crack boundaries prior to crack opening. In fact, the existence of such bridging has been partially considered in recent studies [19], and our results provide direct experimental evidence that may stimulate further development of solidification cracking theories and microstructural simulations such as phase-field modeling. The second feature is that solidification cracks do not form as a single line approximately parallel to the weld centerline but rather as multiple cracks distributed in the direction perpendicular to the weld line, as clearly shown in Frame Nos. 26 and 28. This result suggests that solidification cracking can occur not only strictly in the final solidification region (that is, the meeting point of grains or dendrites growing from both sides of the weld centerline) but also in the surrounding regions. Although rarely reported systematically in previous studies, such a crack morphology provides a new perspective for understanding solidification cracking behavior. Note that these two characteristic behaviors were confirmed throughout the entire crack formation region, not just in the magnified rectangular region in Figure 6.

Based on the above observations, the next section describes the method used to identify the crack initiation timing at each location. The relationships among crack initiation position, temperature, and strain are then quantitatively analyzed.

### 3.3. Quantitative characterization of solidification cracking behavior at the dendrite scale

#### 3.3.1. Identification of crack initiation events and extraction of local conditions

As shown in Figure 7(a), in Case 1, the major solidification crack formed near the weld centerline is defined as the Main branch, whereas the branched cracks extending from it are defined as the Sub branch. The crack initiation timing was identified for all pixels belonging to each branch. Spatial identification of the pixel groups corresponding to the Main and Sub branches was performed based on the crack locations observed in the X-ray transmission images and the associated microstructural features. For the Main branch, regions corresponding to the meeting points of grains or dendrites growing from both sides of the weld centerline were used, whereas for the Sub branch, grain boundary regions between approximately parallel-growing dendrites were used as references. The crack initiation timing was determined from the rapid development of dark regions in the X-ray transmission images during crack formation (Figure 6). To quantify this behavior, the difference in local image-intensity variability between the target location and the surrounding regions was evaluated.

**Figure 7.**
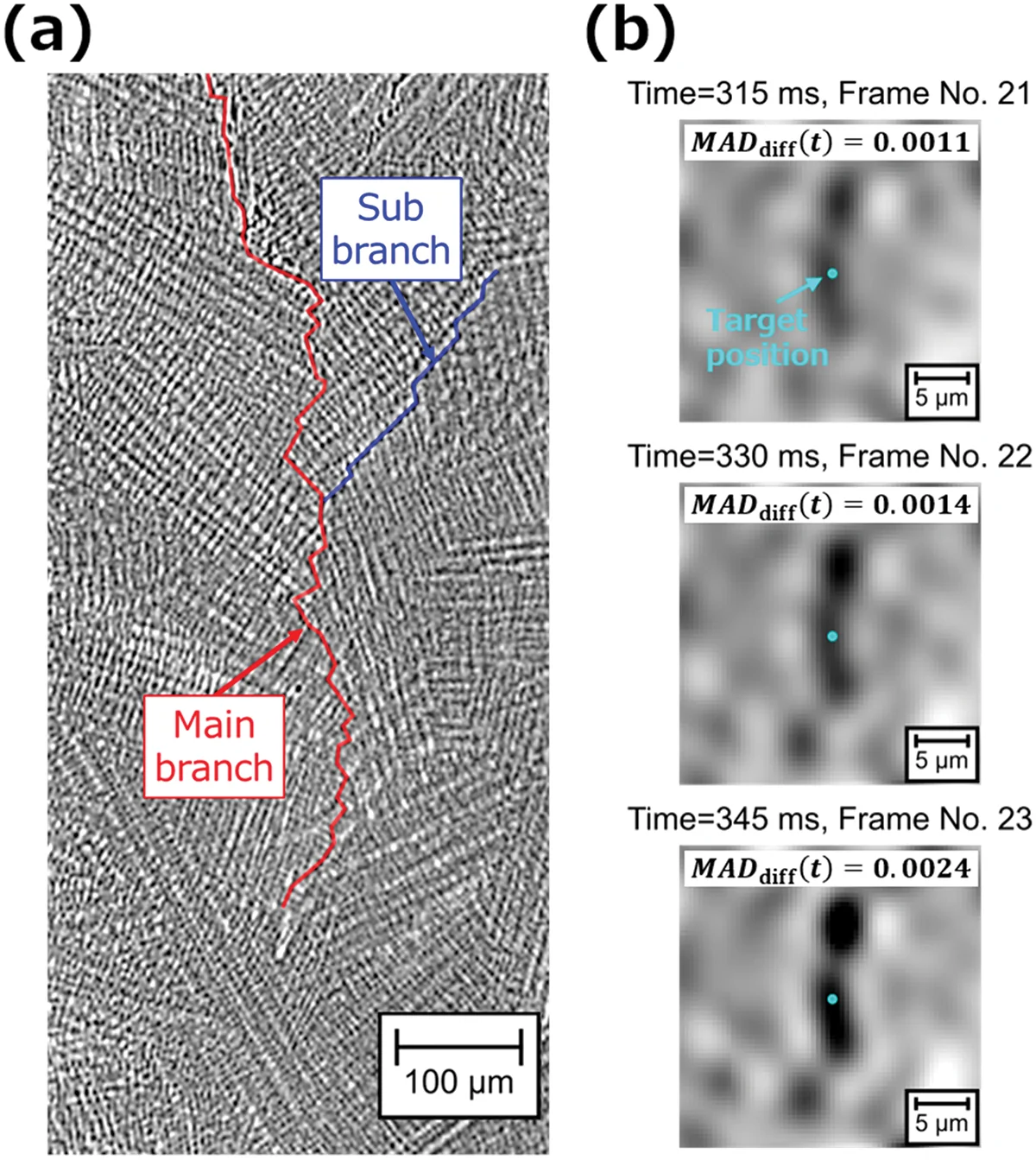
Definition of crack regions and temporal evolution of the crack initiation indicator. (a) Spatial definition of the crack regions in Case 1. The crack formed near the weld centerline is defined as the Main branch, while the branched cracks extending from it are defined as the Sub branch. (b) Representative temporal evolution of the indicator $\mathrm{MA}D_{\mathrm{diff}}$, defined as the difference between the local MAD and the first quartile of surrounding MAD values. Crack initiation is identified when $\mathrm{MA}D_{\mathrm{diff}}$ exceeds the threshold value of 0.002, corresponding to the development of low-brightness regions associated with crack opening.

First, to retain a wider intensity range than that used for strain evaluation, the original time-series images were processed by clipping pixel intensities exceeding ±10σ from the mean intensity to the corresponding threshold values, where σ denotes the standard deviation of the intensity distribution for each image sequence. Denoising (corresponding to the upper procedure in Figure 2(a)) and contrast correction (the middle procedure in Figure 2(a)) were then applied, and the resulting images were converted into inverted grayscale images. For each pixel in the defined branch, a square region of 64 × 64 pixels centered on the pixel was defined. The median absolute deviation (MAD) of the intensity distribution within this region at time t, denoted as $\mathrm{MA}D_{\mathrm{center}}\left(t\right)$, was calculated. In addition, all neighboring 64 × 64 pixel regions adjacent to the central region (a total of 509 regions) were defined as the surrounding regions, and their MAD values were calculated as $\left\{\mathrm{MA}D_{\mathrm{around},i}\left(t\right){\}}_{i=1}^{509}\right.$ The representative difference in intensity variability between the target and surrounding regions, $\mathrm{MA}D_{\mathrm{diff}}\left(t\right)$, was defined as

(8) $$\mathrm{MA}D_{\mathrm{diff}}\left(t\right)=\mathrm{MA}D_{\mathrm{center}}\left(t\right)-Q_{1}\left({\left\{\mathrm{MA}D_{\mathrm{around}},i\left(t\right)\right\}}_{i=1}^{509}\right),$$

where $Q_{1}$ denotes the first quartile. Using this representative value, the influence of noise in the transmission images can be suppressed while evaluating the extent to which the local intensity variability exceeds that of the surrounding regions. In this study, the crack initiation time, $t_{\mathrm{crack}}$, was defined as the time at which $\mathrm{MA}D_{\mathrm{diff}}\left(t\right)$ exceeded a predefined threshold. Because $t_{\mathrm{crack}}$ varies spatially, it is expressed as a function of position, $t_{\mathrm{crack}}\left(x,y\right)$, where the top-left corner of the image is defined as the origin, and the horizontal and vertical directions are defined as x and y, respectively. If $\mathrm{MA}D_{\mathrm{diff}}\left(t\right)$ temporarily exceeded the threshold due to noise and then fell below it, the subsequent time at which the threshold was exceeded again was adopted as $t_{\mathrm{crack}}$. The baseline threshold was set to 0.002 based on comparison with the time-series X-ray transmission images. Figure 7(b) shows the temporal evolution of $\mathrm{MA}D_{\mathrm{diff}}\left(t\right)$ at a representative position. When $\mathrm{MA}D_{\mathrm{diff}}\left(t\right)$ exceeded approximately 0.002, dark regions associated with crack opening developed rapidly, accompanied by the appearance of surrounding bright regions considered to result from redistribution of the residual liquid. In the following analysis, the equivalent strain at crack initiation is defined as the critical equivalent strain, $\varepsilon_{\mathrm{crit}}$, and is hereafter referred to simply as the critical strain. The critical strain $\varepsilon_{\mathrm{crit}}\left(x,y\right)$ and critical temperature $T_{\mathrm{crit}}\left(x,y\right)$ for each pixel were extracted using $t_{\mathrm{crack}}\left(x,y\right)$, together with the time-series equivalent-strain field $\varepsilon_{\mathrm{eq}}\left(x,y,t\right)$ and temperature field $T\left(x,y,t\right)$ (examples shown in Figure 4), as

$$\varepsilon_{\mathrm{crit}}\left(x,y\right)=\frac{\varepsilon_{\mathrm{eq}}\left(x,y,t_{\mathrm{crack}}\left(x,y\right)\right)+\varepsilon_{\mathrm{eq}}\left(x,y,t_{c\mathrm{rack}}^{pre}\left(x,y\right)\right)}{2},$$

(9) $$T_{\mathrm{crit}}\left(x,y\right)=\frac{T\left(x,y,t_{\mathrm{crack}}\left(x,y\right)\right)+T\left(x,y,t_{c\mathrm{rack}}^{pre}\left(x,y\right)\right)}{2},$$

where $t_{cr\mathrm{ack}}^{pre}$ denotes the time one frame before $t_{\mathrm{crack}}$. Thus, the critical equivalent strain and critical temperature were defined as the averages of the corresponding values at the frame in which crack initiation was detected and the immediately preceding frame. To evaluate sensitivity to the temporal resolution of crack-initiation detection, the extraction time was shifted by −1, 0, and +1 frame relative to the baseline time. When the frame one step later was used, the critical temperature decreased by an average of 16.8°C and the critical strain increased by an average of 0.00429. Conversely, when the frame one step earlier was used, the critical temperature increased by an average of 15.8°C and the critical strain decreased by an average of 0.00428. Thus, a shift of ±1 frame produced changes of approximately ±16°C in critical temperature and ±0.0043 in critical strain. These values represent the typical variation in the locally extracted critical temperature and critical strain associated with an uncertainty of one frame in the crack initiation time.

Figure 8 shows the relationship between the critical temperature and critical equivalent strain extracted for the Main and Sub branches. The temperature dependence of the total solid fraction, $f_{s}$, obtained from a Scheil–Gulliver calculation for the FMS alloy composition is also shown. The calculation was performed using Thermo-Calc 2026b and the TCFE14 Steels/Fe-alloys Database. The Scheil–Gulliver calculation predicted austenite (γ phase; FCC_A1 in the Thermo-Calc database) as the primary solid phase. Several additional phases were predicted in extremely small amounts near the final stage of solidification. Because their contributions to the total solid fraction were negligible, the formation of the individual minor phases was not discussed further. Instead, the solidification state was evaluated based on the total solid fraction, defined as the sum of all solid phases, and the residual liquid fraction. Because non-equilibrium solidification accompanied by segregation occurs in the crack-formation region, the equilibrium solidus temperature of approximately 1300°C alone cannot adequately represent the actual solid–liquid coexistence temperature range. The Scheil–Gulliver calculation indicated that residual liquid persisted to temperatures below this equilibrium solidus temperature, thereby extending the effective solid–liquid coexistence region toward lower temperatures. This result is consistent with the previously reported decrease in the final solidification temperature caused by segregation in this alloy [16].

**Figure 8.**
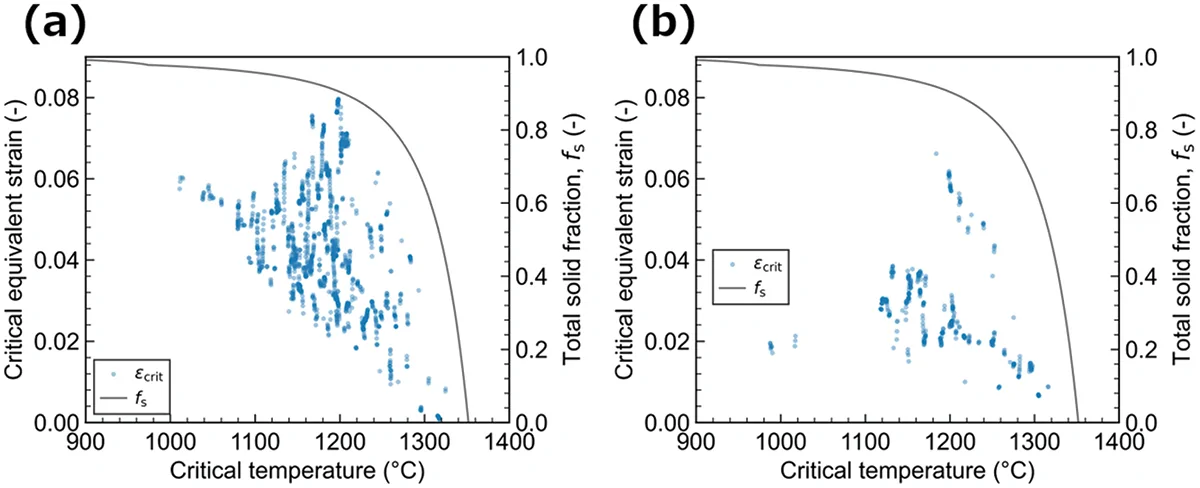
Relationship between critical temperature and critical equivalent strain at crack initiation for (a) the Main branch and (b) the Sub branch. Each point represents the local condition at crack initiation identified from the in-situ observations. The solid line shows the total solid fraction, $f_{s}$, calculated using the Scheil–Gulliver model for the FMS alloy composition with Thermo-Calc 2026b and the TCFE14 steels/Fe-alloys database.

As shown in Figure 8, solidification cracking occurred over a temperature range from approximately 1320 to 1000°C in both branches. According to the Scheil–Gulliver calculation, this range corresponds to an increase in the total solid fraction, $f_{s}$, from approximately 0.5 to 0.97. This result is consistent with the conventional understanding that solidification cracking occurs under solid–liquid coexistence conditions in the presence of residual liquid. In both branches, the critical strain also exhibited substantial scatter at the same or similar critical temperatures. Thus, the Main and Sub branches shared the qualitative feature of a broad critical-strain distribution within the same temperature range. This result indicates that the critical strain at solidification-crack initiation cannot be uniquely determined by temperature alone.

To evaluate the sensitivity of these results to the MAD-difference threshold, the critical temperature and critical strain were also extracted using thresholds of 0.0015 and 0.0025, in addition to the baseline threshold of 0.0020. Supplementary Figure S1(a) and (b) compare the critical temperature–critical equivalent strain relationships obtained for the Main and Sub branches, respectively, using the three thresholds. Although the extracted values varied locally with threshold selection, the overall temperature dependence and the broad critical-strain distributions observed for both branches were maintained.

#### 3.3.2. Data-driven characterization of critical strain depending on temperature and spatial variability

As shown in the preceding section, the critical strain exhibited substantial scatter at the same or similar critical temperatures for both the Main and Sub branches, suggesting that factors other than temperature also influenced the critical strain. Possible contributing factors include the local thermal history, temperature gradient, cooling rate, mechanical constraint, solid–liquid interface morphology, dendrite-bridging condition, solute segregation, and local microstructural arrangement. However, these factors were not measured independently in the present study, and their individual contributions therefore could not be separated. Accordingly, without attributing the observed variation to any specific factor, the critical-strain distribution was first characterized as a function of temperature. The residual obtained by subtracting the corresponding temperature-bin median from the critical strain at each position was then evaluated along the crack path to examine the spatial variation that could not be explained by temperature alone.

To evaluate the temperature-dependent distribution of critical strain, the Main- and Sub-branch data were combined rather than analyzed separately, with the aim of characterizing the overall distribution in Case 1. Comparison of the critical temperature–critical strain relationships for the two branches in Figure 8 showed a small number of relatively high values for the Main branch and relatively low values for the Sub branch near approximately 1000°C, where the number of observations was limited. At other temperatures, however, the distributions of the two branches largely overlapped. Therefore, combining the data from both branches was considered appropriate for evaluating the overall distributional characteristics of Case 1. Specifically, the combined data were divided into 30°C-wide bins according to the critical temperature. The median, 25th and 75th percentiles, and 10th and 90th percentiles of the critical strain were calculated for each temperature bin. Bins containing fewer than 30 pixel-level observations were excluded from the percentile representation to avoid unstable percentile estimates in sparsely populated bins. This threshold was introduced solely as a descriptive criterion based on data density and does not establish statistical independence among neighboring observations. Therefore, the number of pixels in each bin should not be interpreted as the number of independent specimens or independent samples.

The sensitivity of the temperature-binned results to the MAD-difference threshold was also examined. Supplementary Figure S1(c) compares the median critical strain obtained from the combined Main- and Sub-branch data using thresholds of 0.0015, 0.0020, and 0.0025, together with the 10th–90th percentile range for the baseline threshold of 0.0020. Although the median values varied slightly with threshold selection, the overall temperature dependence and the broad distribution within each temperature range were maintained.

Figure 9(a) shows the relationship between critical temperature and critical equivalent strain for the combined Main- and Sub-branch data in Case 1, together with the percentiles calculated directly for each temperature bin. The median represents the central tendency of the critical strain in each temperature range, whereas the 25th–75th and 10th–90th percentile ranges represent the central portion and the broader width of the distribution, respectively. The median critical strain was higher at lower temperatures and generally decreased toward higher temperatures. Thus, under the present experimental conditions, a larger strain was accumulated before crack initiation at the lower-temperature, more advanced stage of solidification. However, this trend should be regarded as the central tendency of the observed distribution rather than as a universal relationship between critical strain and temperature, because crack initiation may also depend on local thermal, mechanical, and microstructural conditions. At the same time, the critical strain within each temperature bin was broadly distributed around the median. A clear width was observed even within the 25th–75th percentile range, while the 10th–90th percentile range extended over a still broader range of values. Thus, the scatter in critical strain was not caused only by a small number of extreme values but was also present within the central portion of the distribution. These results indicate that temperature is a major factor governing the central tendency of critical strain, whereas the critical strain at an individual location cannot be uniquely determined from temperature alone. It should also be noted that the observed distribution may include contributions from both local physical variability and the measurement and extraction procedures. Although the sensitivity analyses indicated that the broad distribution was maintained under the examined detection conditions, the individual contributions of physical variability and measurement-related uncertainty could not be fully separated in the present study.

**Figure 9.**
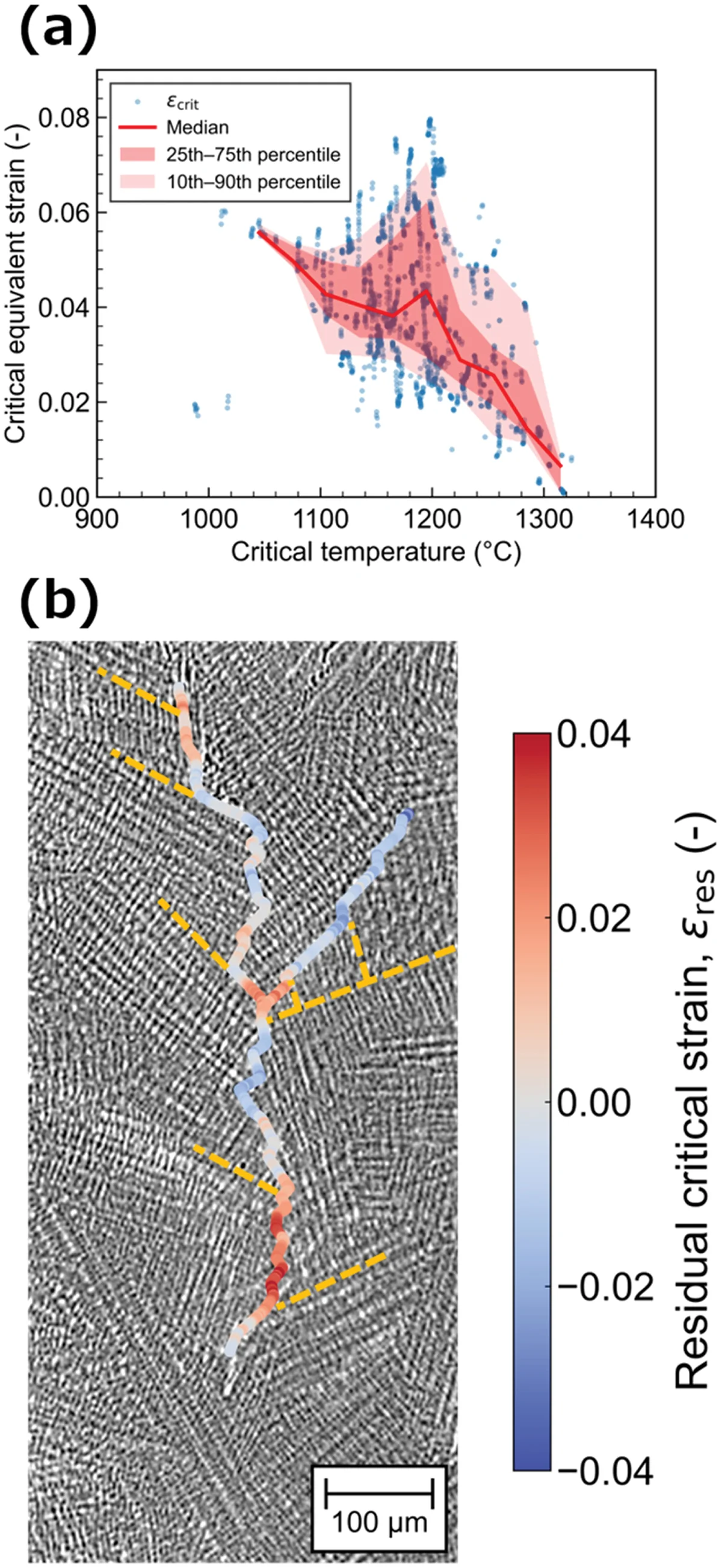
Temperature-dependent distribution and spatial variation of critical strain in Case 1. (a) Relationship between critical temperature and critical equivalent strain for the combined Main and Sub branches. The solid line indicates the median in each 30°C temperature bin, and the dark- and light-shaded regions indicate the 25th–75th and 10th–90th percentile ranges, respectively. (b) Spatial distribution of the critical-strain residual along the crack path overlaid on the X-ray transmission image. The residual was calculated by subtracting the median critical strain of the corresponding temperature bin from the critical strain at each position. The yellow dashed lines indicate grain boundaries adjacent to the crack path.

Next, the spatial variation in critical strain that was not explained by the central temperature-dependent tendency was evaluated. The critical-strain residual, $\varepsilon_{\mathrm{res},i}$, at observation point i was defined by subtracting the median critical strain of the corresponding temperature bin, ${\tilde{\varepsilon }}_{\mathrm{crit},b\left(i\right)}$, from the critical strain at that point, $\varepsilon_{\mathrm{crit},i}$, as follows:

(10) $$\varepsilon_{\mathrm{res},i}=\varepsilon_{\mathrm{crit},i}-{\tilde{\varepsilon }}_{\mathrm{crit},b\left(i\right)}$$

where $b\left(i\right)$ denotes the temperature bin containing observation point i. This residual represents the extent to which the critical strain at each observation point is higher or lower than the median of the corresponding temperature bin. Thus, a positive residual indicates that the critical strain at that position is higher than the bin median, whereas a negative residual indicates that it is lower. This procedure enabled the relative variation in critical strain, after removal of the central temperature-dependent tendency, to be evaluated at each position along the crack path.

Figure 9(b) shows the critical-strain residuals overlaid on the X-ray transmission image along the entire crack path, including both the Main and Sub branches. The residuals changed between positive and negative values depending on position, and regions in which residuals of the same sign or similar magnitude persisted over a finite distance were also observed. In addition, some locations at which the residual changed appeared to correspond to changes in the combination of grains facing each other across the crack. These observations suggest that the variation remaining after subtraction of the temperature-bin median was not spatially uniform and may have been influenced by position-dependent factors, including the local microstructural arrangement. However, the present analysis does not identify the physical origin of this spatial variation. Position along the crack path may be associated with multiple local factors, including the microstructural state, thermal and mechanical conditions, solid–liquid interface morphology, dendrite-bridging condition, and solute segregation. Because these factors were not measured independently, the observed spatial variation in the residual cannot be attributed to a particular grain combination or to any single microstructural factor.

Overall, the critical strain at solidification-crack initiation exhibited a temperature-dependent central tendency together with a broad distribution within each temperature range. The variation remaining after removal of this temperature dependence was spatially nonuniform along the crack path. Although local microstructural arrangements, including the combination of grains facing each other across the crack, may contribute to this variation, their effects could not be separated from those of other position-dependent factors. Thus, the critical strain should be regarded as a temperature-dependent distribution rather than a single-valued curve. Further work is required to identify the physical factors governing the distribution width and its spatial variation.

#### 3.3.3. Comparison with the equivalent-strain distribution under the non-cracking condition

In the preceding section, the critical strain at solidification-crack initiation in Case 1 was shown to exhibit a temperature-dependent central tendency together with a broad distribution within the same temperature range. The residual variation after removal of this temperature dependence was also shown to be spatially nonuniform along the crack path. In this section, the equivalent-strain distribution in Case 2, in which no crack occurred, was compared with the critical-strain distribution obtained for Case 1 to examine their consistency at the distribution level. Because no crack initiation event occurred in Case 2, however, a critical value cannot be defined for this case, and the present comparison should not be regarded as an independent validation of the critical-strain distribution obtained from Case 1.

Figure 10 compares the distribution of critical equivalent strain at crack initiation in Case 1 with the equivalent-strain distribution under the non-cracking condition in Case 2. For Case 1, the median and 10th–90th percentile range of the critical strain obtained for each temperature bin in Figure 9(a) are shown. For Case 2, the equivalent-strain values obtained at all times and positions at temperatures of 1000°C or higher were grouped into the same 30°C temperature bins used for Case 1, and the median, 90th percentile, and 99th percentile were calculated for each bin. The 90th and 99th percentiles were used to characterize the upper part of the Case 2 distribution and to determine how closely the locally high strain values approached the critical-strain distribution observed in Case 1. Overall, the equivalent strain in Case 2 was lower than the critical strain in Case 1. In particular, the median and 90th percentile of Case 2 remained below the 10th percentile of Case 1 over almost the entire temperature range. This result indicates that, at most locations in Case 2, the equivalent strain did not reach even the lower part of the critical-strain distribution observed at crack initiation in Case 1. This overall relationship is consistent with the absence of solidification cracking in Case 2. In contrast, the 99th percentile of Case 2 approached the 10th percentile of Case 1 in some temperature ranges and partially overlapped the 10th–90th percentile range of Case 1. Nevertheless, no solidification cracking was observed in Case 2. This result indicates that the 10th percentile of Case 1 cannot be treated as a deterministic crack-initiation limit governed by temperature alone. In other words, even when the equivalent strain at a certain location reaches the lower part of the critical-strain distribution observed in Case 1, crack initiation does not necessarily occur. The difference between Cases 1 and 2 may have been influenced by differences in local thermal history, temperature gradient, cooling rate, mechanical constraint, strain rate, solid–liquid interface morphology, liquid-film connectivity, dendrite-bridging condition, solute segregation, and local microstructural arrangement. Variations in these local conditions may alter the amount of strain that can be accommodated before crack initiation at the same temperature. However, because these factors were not measured independently in the present study, the difference between the two cases cannot be attributed to any specific factor.

**Figure 10.**
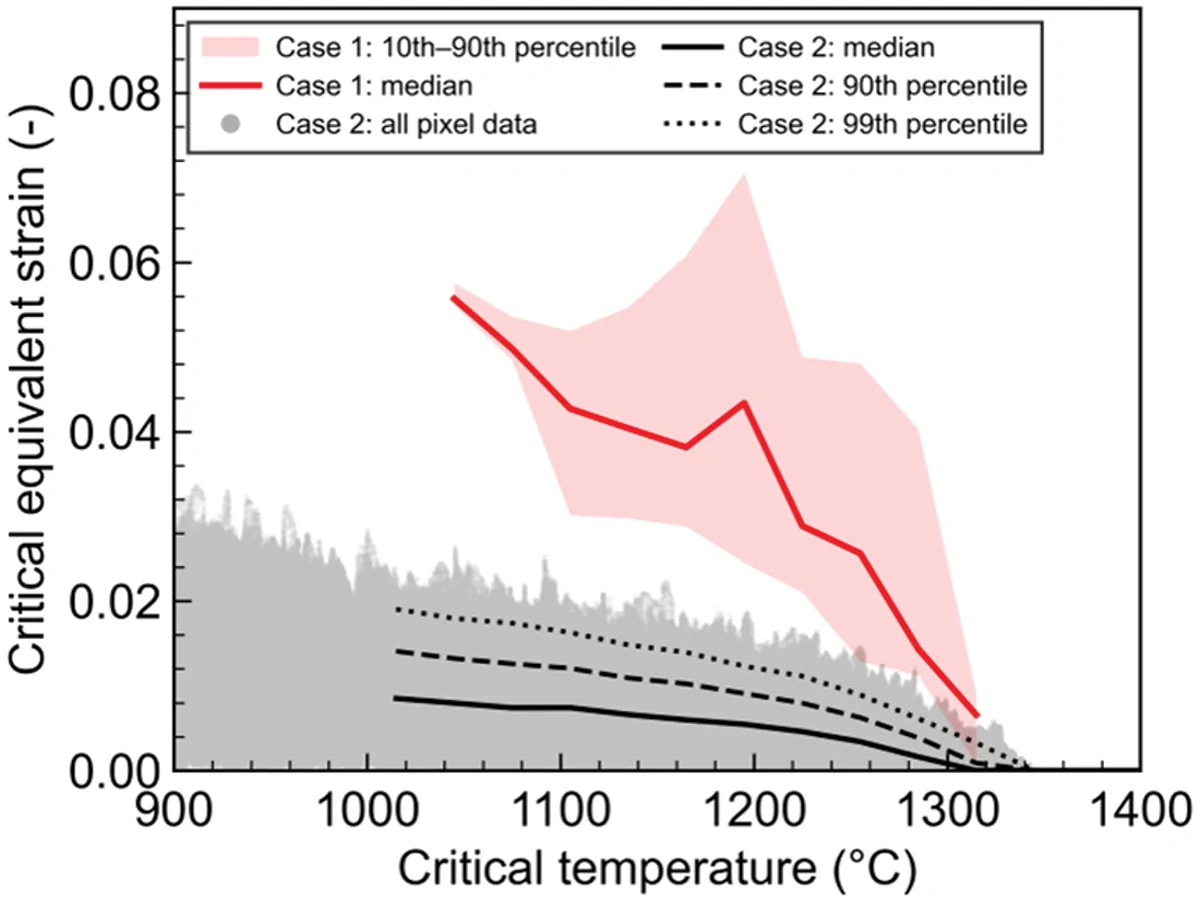
Comparison of the equivalent-strain distributions in cases 1 and 2 as functions of temperature. For Case 1, the solid line and shaded region indicate the median and the 10th–90th percentile range, respectively, of the critical equivalent strain at crack initiation obtained from the combined Main- and Sub-branch data. For Case 2, the gray points represent all pixel-level equivalent-strain data under the non-cracking condition, while the solid, dashed, and dotted lines indicate the median, 90th percentile, and 99th percentile, respectively. The same 30°C temperature bins were used for both cases.

Overall, most of the Case 2 strain distribution lay below the critical-strain distribution in Case 1, which was broadly consistent with the non-cracking condition. However, the upper part of the Case 2 distribution partially overlapped the critical-strain distribution in Case 1. Therefore, the crack-initiation limit cannot be defined as either a single temperature-dependent curve or a particular percentile of the Case 1 distribution. Predicting solidification-crack initiation will require consideration not only of the temperature-dependent critical-strain distribution, but also of a position-dependent crack-initiation limit that varies with the local thermal, mechanical, and microstructural conditions.

### 3.4. Limitations

By combining synchrotron X-ray transmission imaging with analyses of the local strain and temperature fields, the present study showed that the critical strain at solidification-crack initiation exhibited a temperature-dependent central tendency together with a broad distribution within the same temperature range. The critical-strain residuals obtained after removal of the temperature-dependent tendency were also spatially nonuniform along the crack path. In addition, the upper part of the strain distribution under the non-cracking condition in Case 2 partially overlapped the critical-strain distribution obtained in Case 1. Nevertheless, the present study has several limitations.

Firstly, the physical factors governing the width and spatial variation of the critical-strain distribution could not be identified. Local thermal history, temperature gradient, cooling rate, mechanical constraint, strain rate, solid–liquid interface morphology, liquid-film connectivity, dendrite-bridging condition, solute segregation, and local microstructural arrangement were not measured independently. Therefore, the spatial variation in the critical-strain residuals observed in Figure 9(b) cannot be attributed to a particular grain combination or to any single microstructural factor. Future studies should evaluate these local factors independently or simultaneously and examine their relationships with the critical-strain distribution.

Secondly, the extracted critical temperatures and critical strains contain uncertainties associated with the image-processing conditions and the identification of crack-initiation timing. In the present study, sensitivity analyses were performed by varying the threshold used for the MAD difference and by shifting the crack-initiation time by ±1 frame relative to the baseline time. A shift of one frame resulted in changes of approximately ±16°C in critical temperature and ±0.0043 in critical strain. These values indicate the representative variation associated with the temporal resolution of the extraction procedure. However, they do not provide a complete uncertainty budget that includes the temperature field, strain field, image registration, and crack-detection procedure. Future work should quantify these uncertainty sources individually and combine them into an integrated uncertainty assessment.

Thirdly, Cases 1 and 2 were each based on a single observation condition. Moreover, because no crack occurred in Case 2, the critical strain at crack initiation could not be directly defined for that case. Therefore, the comparison shown in Figure 10 does not constitute an independent validation of the critical-strain distribution obtained from Case 1, but rather a distribution-level consistency check against a non-cracking condition. It was also not confirmed that the local constraint and microstructural states were identical between the two cases. Repeated experiments under multiple conditions are therefore required to evaluate the generality of the observed distributional relationship.

Fourthly, the critical-strain distribution obtained in this study may depend on the investigated Fe–Mn–Cr–Ni–Si alloy, specimen geometry, welding conditions, and observation conditions. Solidification-cracking susceptibility varies with alloy composition, segregation behavior, solidification microstructure, thermal history, and mechanical constraint. Consequently, the temperature-dependent distribution obtained here cannot be directly transferred to other materials or processing conditions. Similar analyses should therefore be conducted for different alloy compositions and welding conditions to distinguish common characteristics from material- and condition-specific behavior.

Despite these limitations, the present study provides experimental evidence that the critical strain at solidification-crack initiation is better represented as a temperature-dependent distribution than as a single-valued curve. The observed finite distribution width and spatially nonuniform residual variation provide a basis for considering local, position-dependent crack-initiation limits. Future work combining quantitative measurements of local thermal, mechanical, and microstructural conditions with repeated in-situ observations will be required to identify the factors governing this distribution and to establish a predictive framework.

## 4. Conclusions

We have investigated the dendrite-scale distribution of critical strain associated with solidification-crack initiation in an Fe–Mn–Cr–Ni–Si alloy by combining synchrotron X-ray transmission imaging with local strain and temperature analyses. The principal findings are summarized as follows.

1. In-situ X-ray imaging revealed the development of a major crack near the weld centerline, together with branched cracks and local solid bridges formed across the crack path. These observations demonstrated that crack propagation was spatially heterogeneous at the dendrite scale.
2. A local image-variability method based on the median absolute deviation was developed to identify the crack-initiation time at each position along the Main and Sub branches. The corresponding critical equivalent strain and critical temperature were then extracted from the time-resolved strain and temperature fields. Sensitivity analyses showed that a shift of one frame in the detected crack-initiation time produced typical variations of approximately ±16°C in critical temperature and ±0.0043 in critical strain.
3. Solidification-crack initiation occurred over approximately 1320–1000°C. The Scheil–Gulliver calculation indicated that this range corresponded to an increase in total solid fraction from approximately 0.5 to 0.97 and that residual liquid persisted below the equilibrium solidus temperature.
4. The median critical strain was higher at lower temperatures and generally decreased toward higher temperatures. However, a broad critical-strain distribution remained within each 30°C temperature range, including a clear width in the central 25th–75th percentile range. Thus, temperature governed the central tendency of critical strain but did not uniquely determine the critical strain at an individual location.
5. After subtraction of the temperature-bin median, the remaining critical-strain variation was spatially nonuniform along the crack path. Although local microstructural arrangements may have contributed to this variation, their effects could not be separated from those of other local thermal, mechanical, and solidification-related factors.
6. Under the non-cracking condition of Case 2, most of the equivalent-strain distribution remained below the critical-strain distribution obtained in Case 1. However, the upper tail of the Case 2 distribution partially overlapped the lower part of the Case 1 distribution. Therefore, neither a single temperature-dependent curve nor a particular percentile of the Case 1 distribution can be regarded as a deterministic crack-initiation limit.

These results provide experimental evidence that the critical strain at solidification-crack initiation is better represented as a temperature-dependent distribution than as a single-valued curve. Further measurements of local thermal, mechanical, and microstructural conditions, together with repeated in-situ observations, will be required to identify the physical factors governing the distribution width and its spatial variation and to establish a predictive framework for position-dependent crack-initiation limits.

## Supplementary Material

Supplemental Material

## Data Availability

The data that support the findings of this study are available from the corresponding author upon reasonable request.
