# Supplementary material for: Dendrite-scale quantitative evaluation of solidification cracking in welding using in-situ synchrotron X-ray imaging: distribution characteristics of critical strain
Source: Sci Technol Adv Mater. 2026 Oct 12;27(1):2736326. doi: 10.1080/14686996.2026.2736326 (PMC13686218; doi:10.1080/14686996.2026.2736326)
Supplement: Supplementary file 1 [file TSTA_A_2736326_SM8346.docx]

Figure S1. Sensitivity of the extracted critical conditions to the MAD-difference threshold.

(a, b) Relationships between critical temperature and critical equivalent strain extracted using MAD-difference thresholds of 0.0015, 0.0020, and 0.0025 for the Main and Sub branches, respectively. (c) Temperature-binned median critical strain calculated from the combined Main- and Sub-branch data for each threshold. The shaded region indicates the 10th–90th percentile range obtained using the baseline threshold of 0.0020. Although the extracted values varied locally with threshold selection, the overall temperature dependence and the broad critical-strain distribution within each temperature range were maintained.
